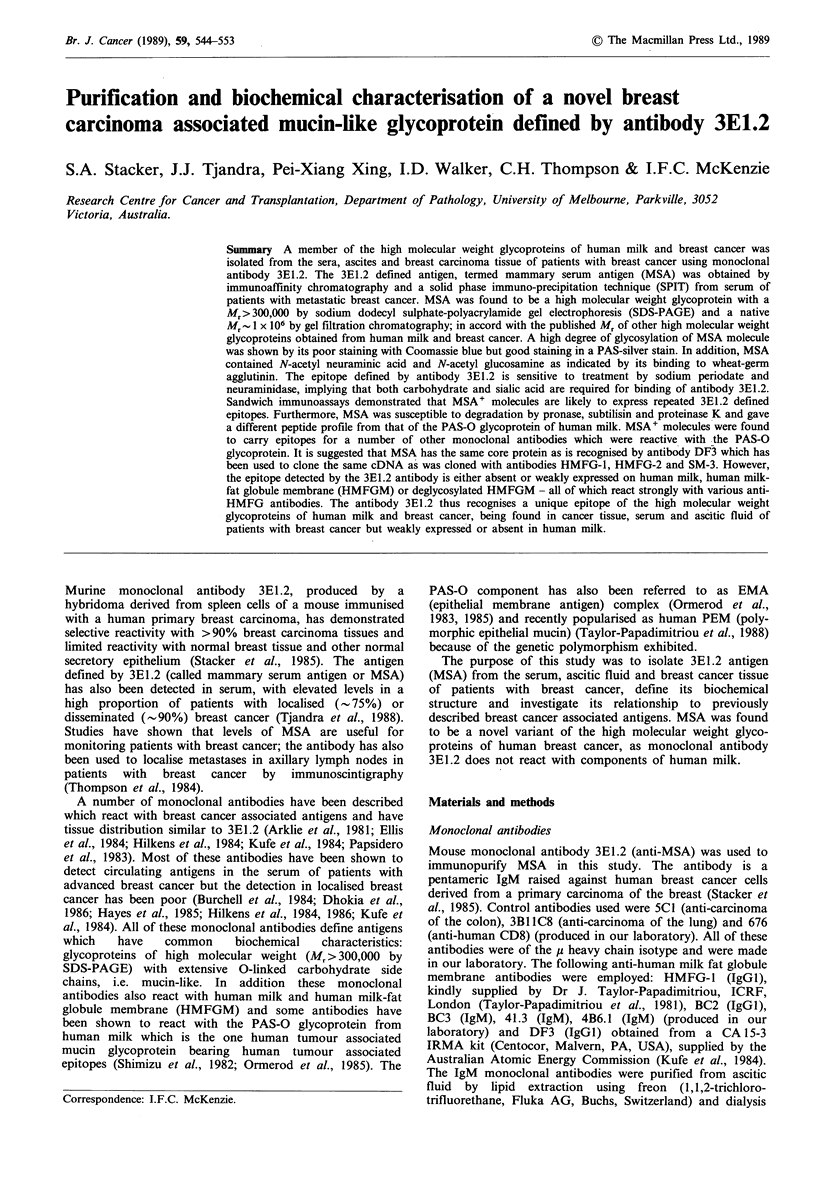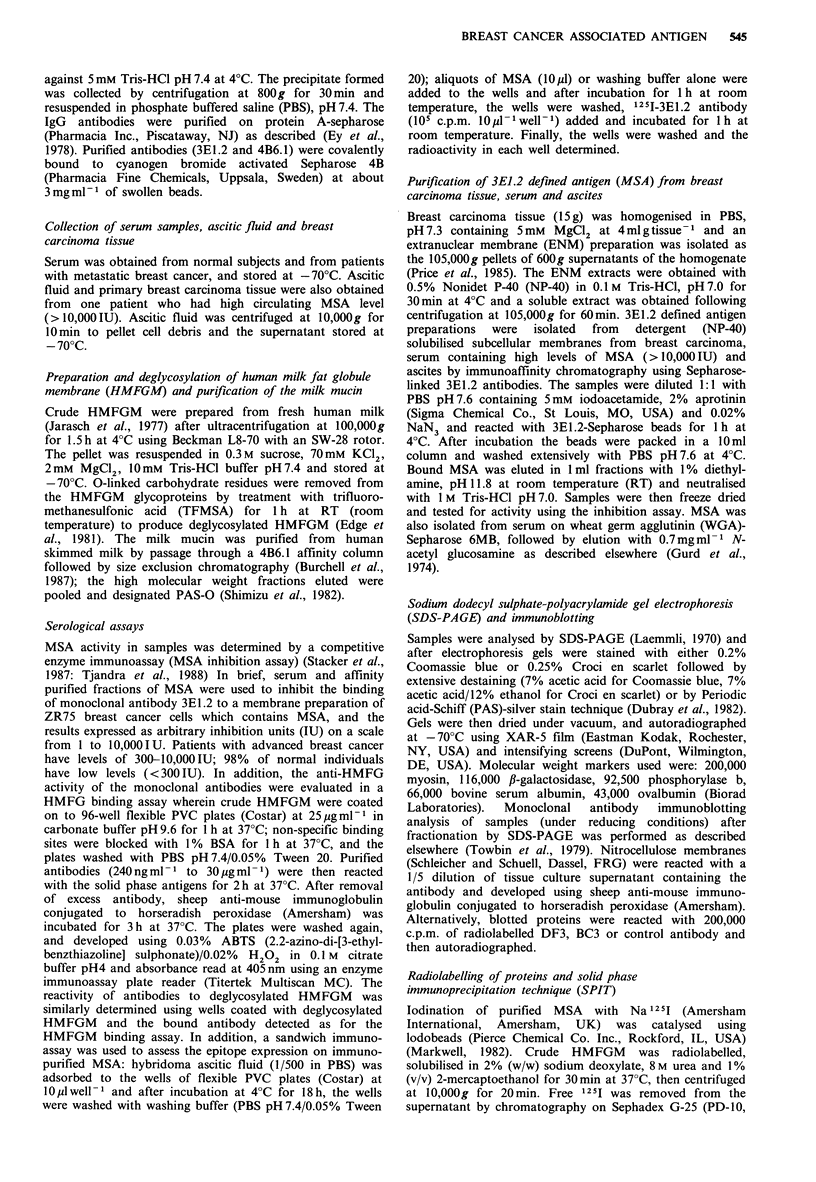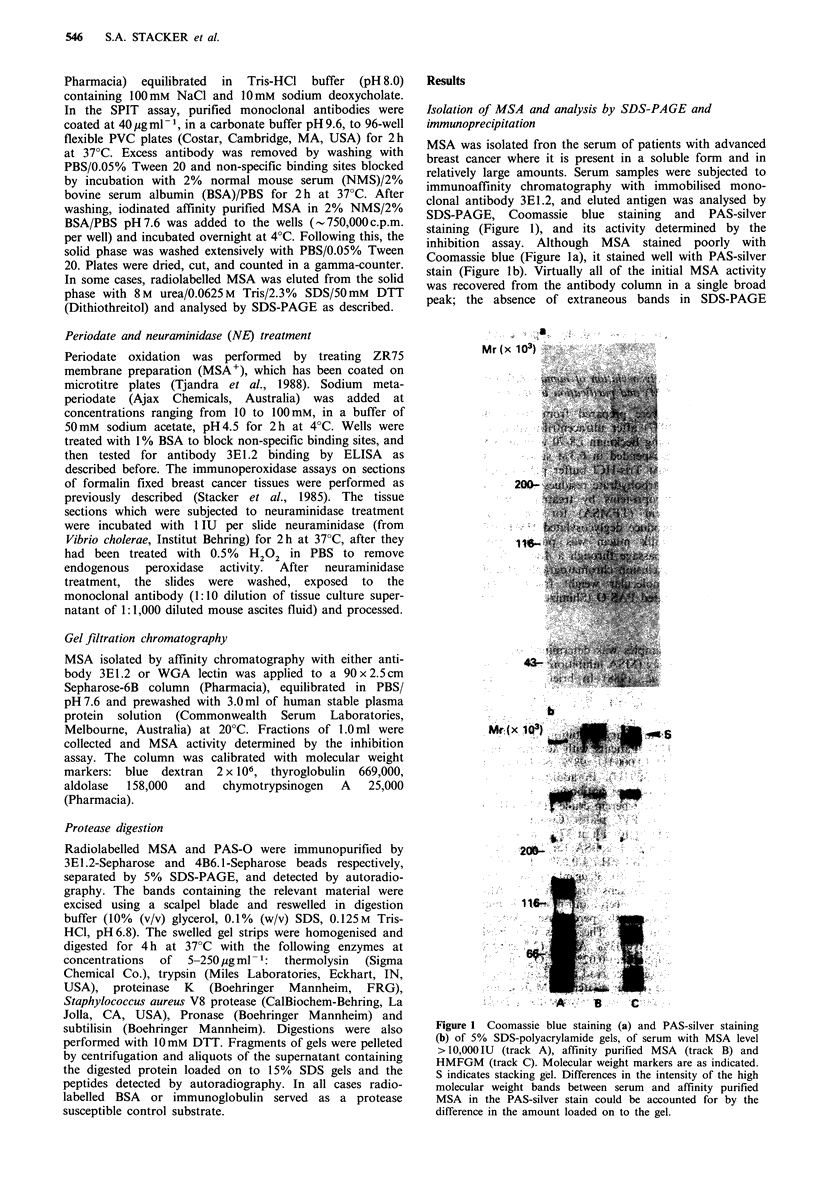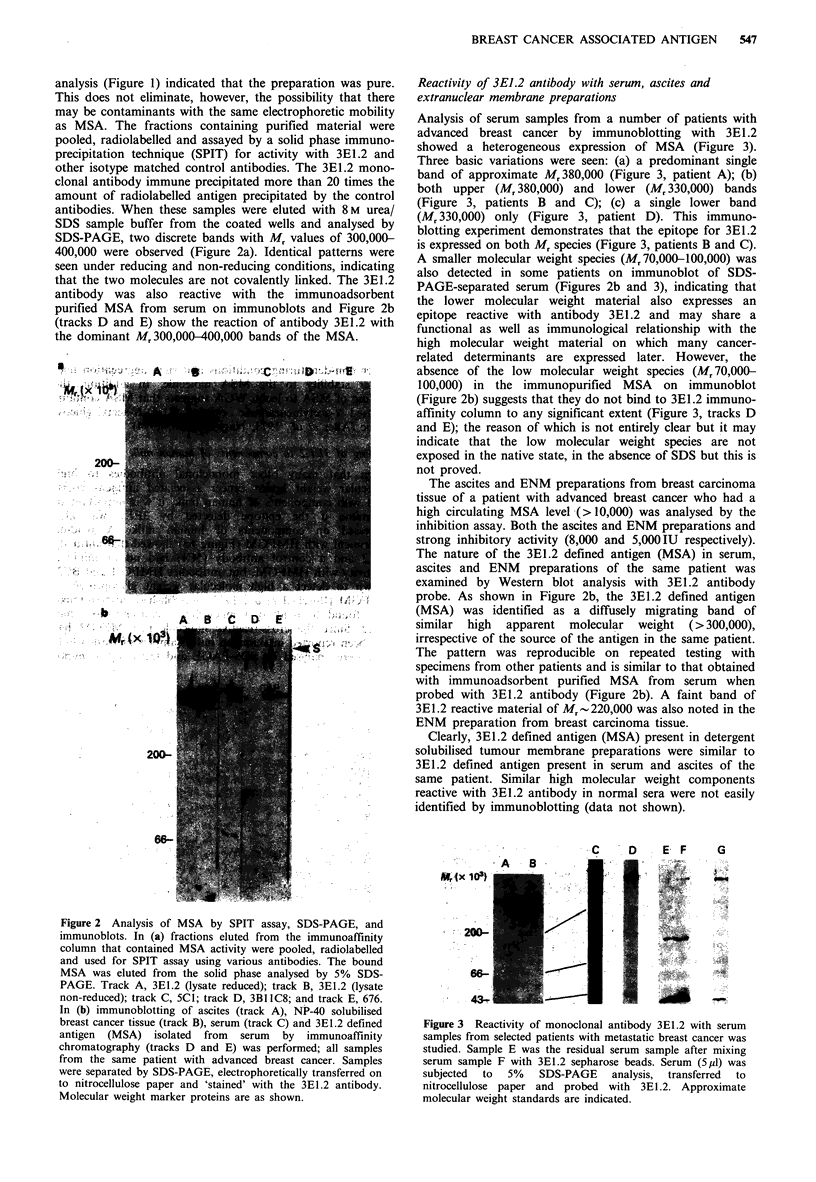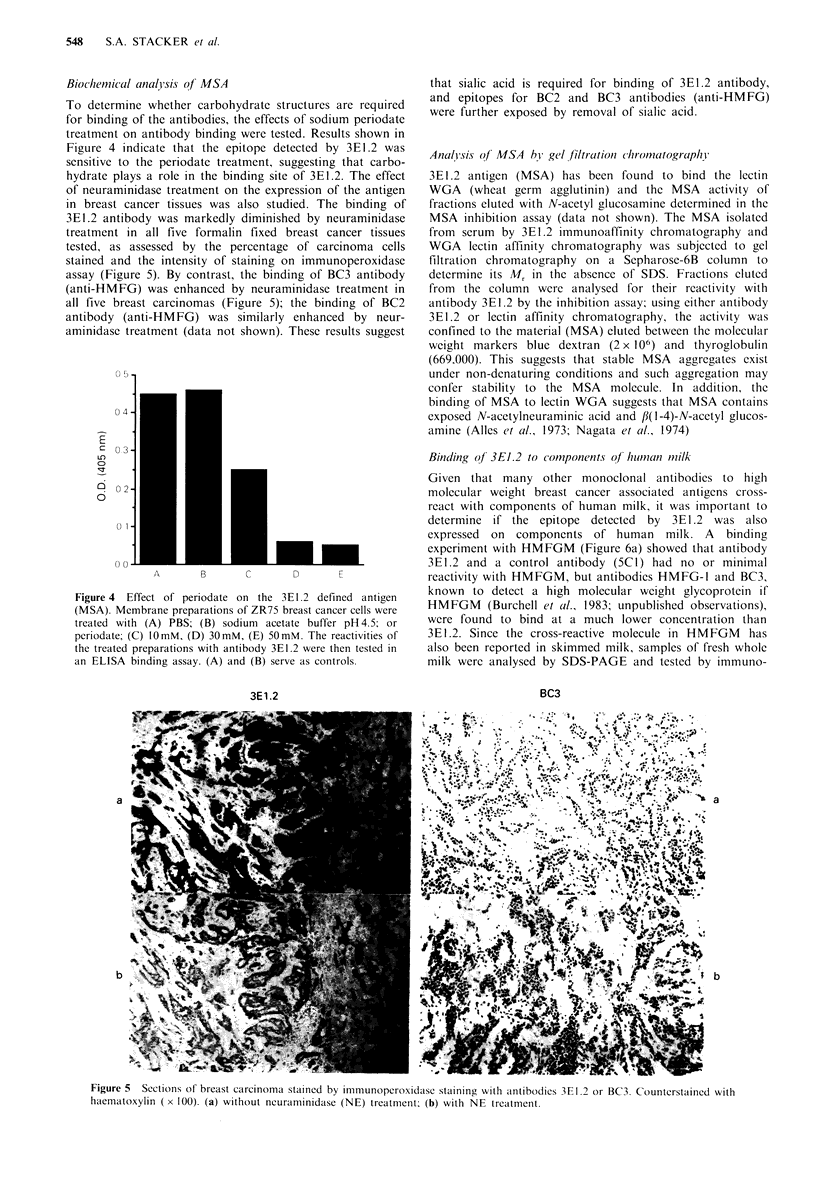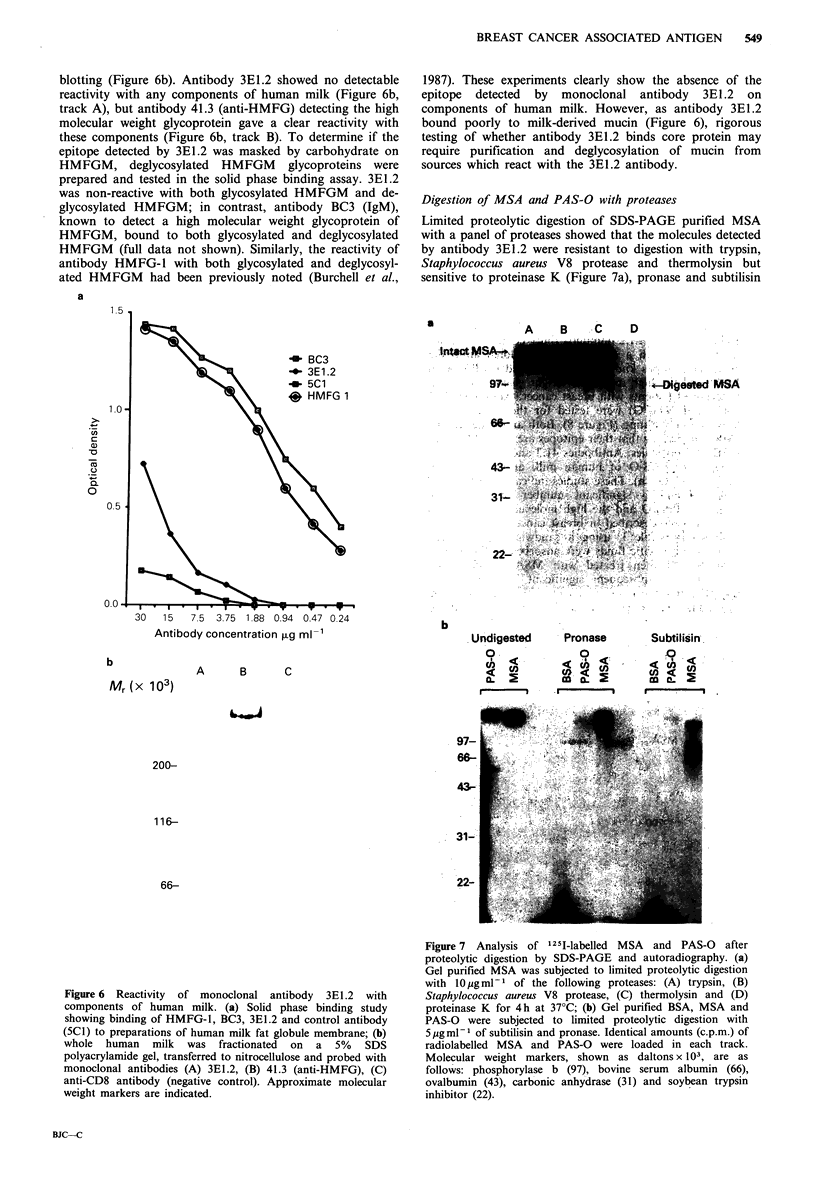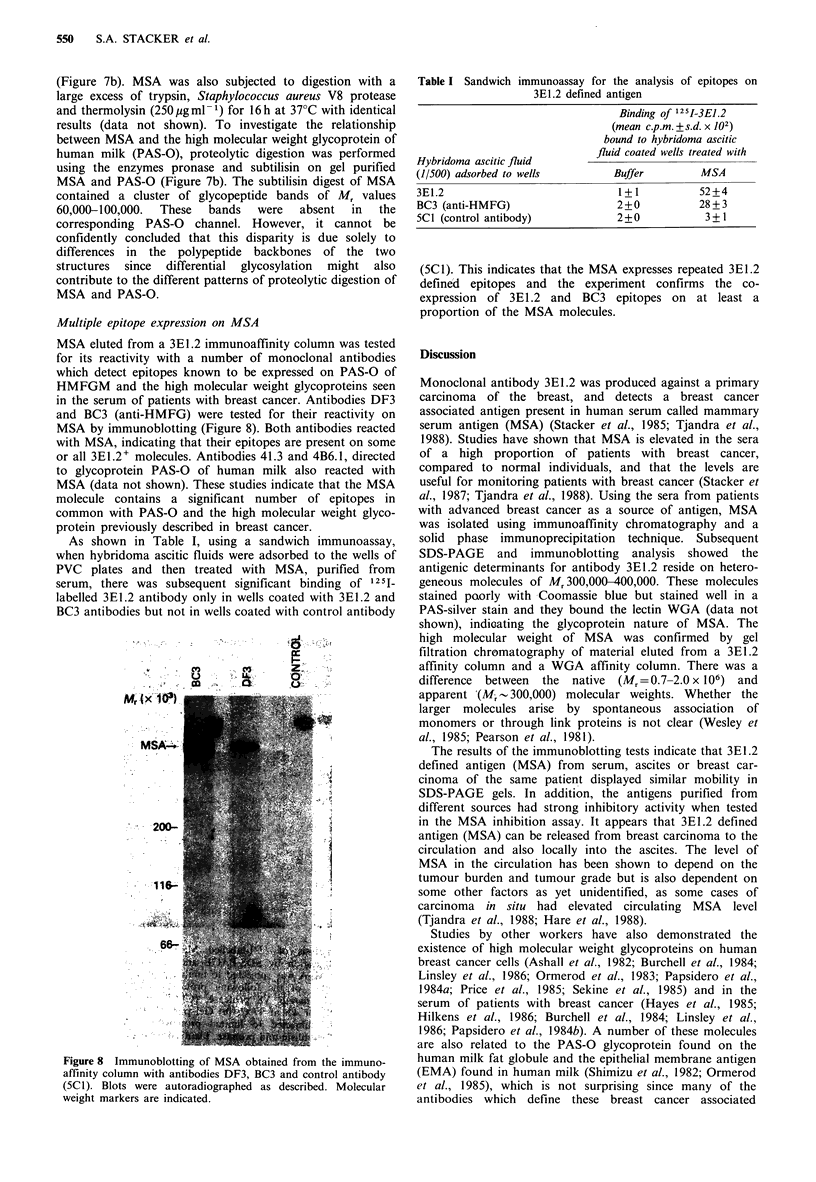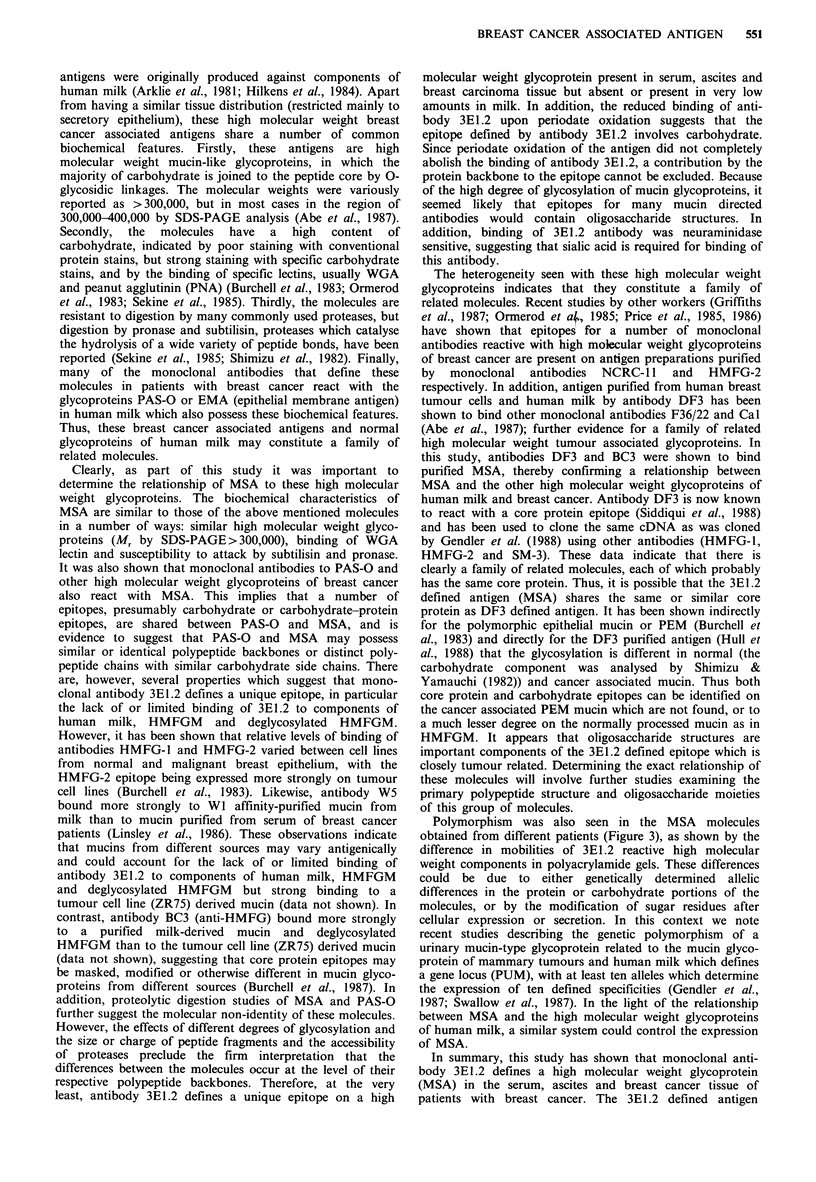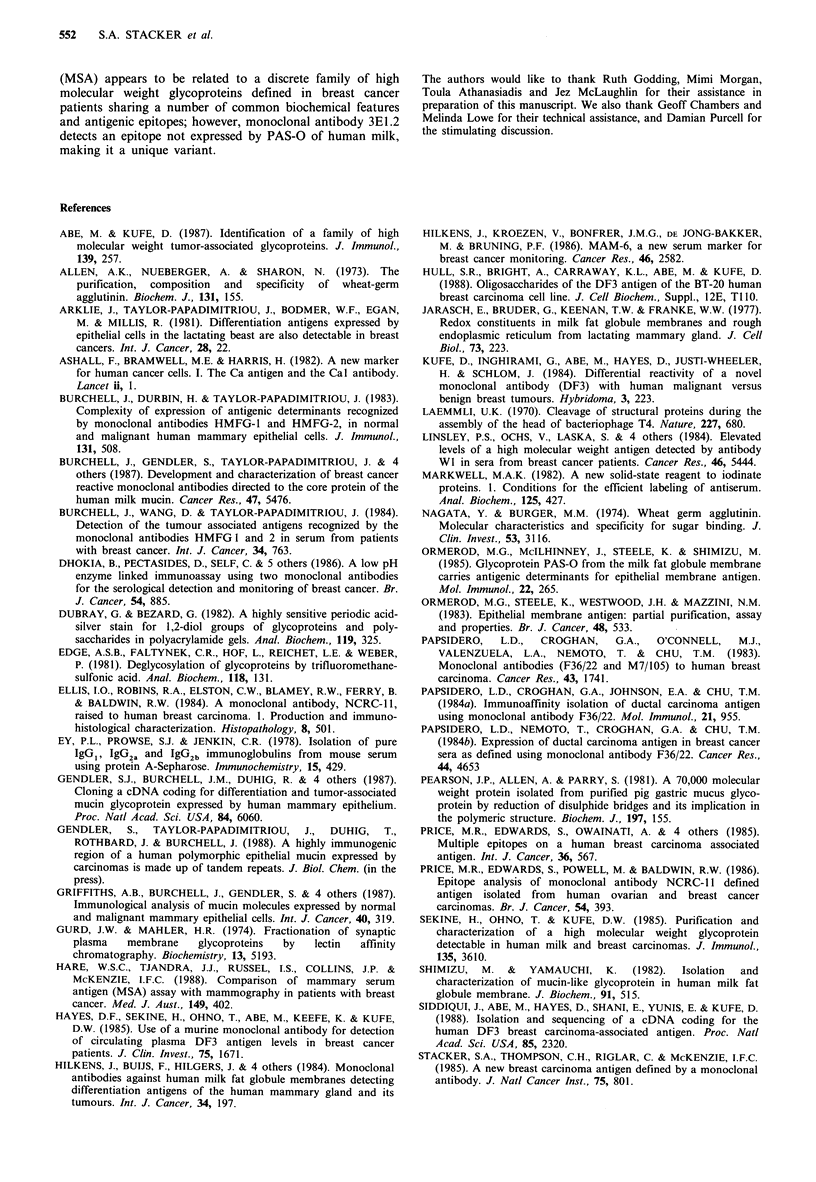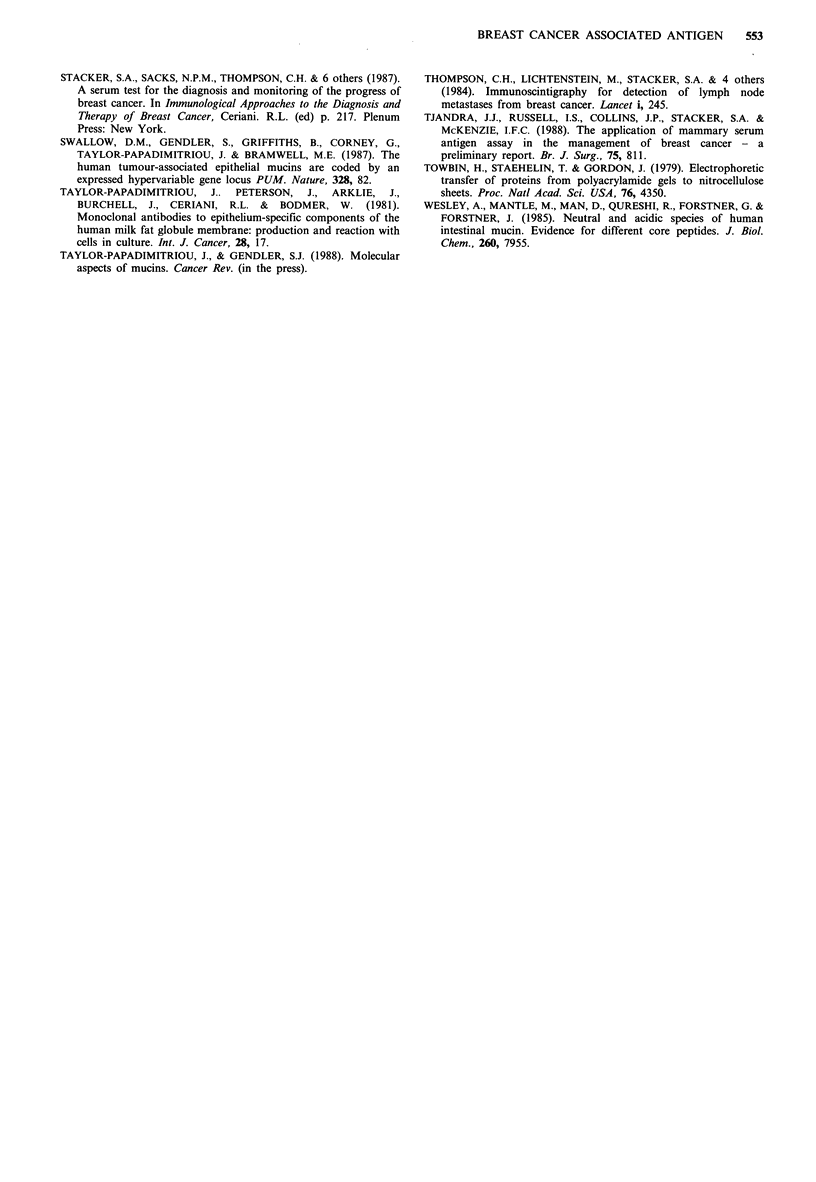# Purification and biochemical characterisation of a novel breast carcinoma associated mucin-like glycoprotein defined by antibody 3E1.2.

**DOI:** 10.1038/bjc.1989.111

**Published:** 1989-04

**Authors:** S. A. Stacker, J. J. Tjandra, P. X. Xing, I. D. Walker, C. H. Thompson, I. F. McKenzie

**Affiliations:** Department of Pathology, University of Melbourne, Victoria, Australia.

## Abstract

**Images:**


Br. J. Cancer (1989), 59, 544–553 © The Macmillan Press Ltd., 1989

### Purification and biochemical characterisation of a novel breast carcinoma associated mucin-like glycoprotein defined by antibody 3E1.2

S.A. Stacker, J.J. Tjandra, Pei-Xiang Xing, I.D. Walker, C.H. Thompson & I.F.C. McKenzie

*Research Centre for Cancer and Transplantation, Department of Pathology, University of Melbourne, Parkville, 3052 Victoria, Australia.*

**Summary** A member of the high molecular weight glycoproteins of human milk and breast cancer was isolated from the sera, ascites and breast carcinoma tissue of patients with breast cancer using monoclonal antibody 3E1.2. The 3E1.2 defined antigen, termed mammary serum antigen (MSA) was obtained by immunoaffinity chromatography and a solid phase immuno-precipitation technique (SPIT) from serum of patients with metastatic breast cancer. MSA was found to be a high molecular weight glycoprotein with a $M_r > 300,000$ by sodium dodecyl sulphate-polyacrylamide gel electrophoresis (SDS-PAGE) and a native $M_r \sim 1 \times 10^6$ by gel filtration chromatography; in accord with the published $M_r$ of other high molecular weight glycoproteins obtained from human milk and breast cancer. A high degree of glycosylation of MSA molecule was shown by its poor staining with Coomassie blue but good staining in a PAS-silver stain. In addition, MSA contained *N*-acetyl neuraminic acid and *N*-acetyl glucosamine as indicated by its binding to wheat-germ agglutinin. The epitope defined by antibody 3E1.2 is sensitive to treatment by sodium periodate and neuraminidase, implying that both carbohydrate and sialic acid are required for binding of antibody 3E1.2. Sandwich immunoassays demonstrated that $MSA^+$ molecules are likely to express repeated 3E1.2 defined epitopes. Furthermore, MSA was susceptible to degradation by pronase, subtilisin and proteinase K and gave a different peptide profile from that of the PAS-O glycoprotein of human milk. $MSA^+$ molecules were found to carry epitopes for a number of other monoclonal antibodies which were reactive with the PAS-O glycoprotein. It is suggested that MSA has the same core protein as is recognised by antibody DF3 which has been used to clone the same cDNA as was cloned with antibodies HMFG-1, HMFG-2 and SM-3. However, the epitope detected by the 3E1.2 antibody is either absent or weakly expressed on human milk, human milk-fat globule membrane (HMFGM) or deglycosylated HMFGM – all of which react strongly with various anti-HMFG antibodies. The antibody 3E1.2 thus recognises a unique epitope of the high molecular weight glycoproteins of human milk and breast cancer, being found in cancer tissue, serum and ascitic fluid of patients with breast cancer but weakly expressed or absent in human milk.

Murine monoclonal antibody 3E1.2, produced by a hybridoma derived from spleen cells of a mouse immunised with a human primary breast carcinoma, has demonstrated selective reactivity with >90% breast carcinoma tissues and limited reactivity with normal breast tissue and other normal secretory epithelium (Stacker *et al.*, 1985). The antigen defined by 3E1.2 (called mammary serum antigen or MSA) has also been detected in serum, with elevated levels in a high proportion of patients with localised (~75%) or disseminated (~90%) breast cancer (Tjandra *et al.*, 1988). Studies have shown that levels of MSA are useful for monitoring patients with breast cancer; the antibody has also been used to localise metastases in axillary lymph nodes in patients with breast cancer by immunoscintigraphy (Thompson *et al.*, 1984).

A number of monoclonal antibodies have been described which react with breast cancer associated antigens and have tissue distribution similar to 3E1.2 (Arklie *et al.*, 1981; Ellis *et al.*, 1984; Hilkens *et al.*, 1984; Kufe *et al.*, 1984; Papsidero *et al.*, 1983). Most of these antibodies have been shown to detect circulating antigens in the serum of patients with advanced breast cancer but the detection in localised breast cancer has been poor (Burchell *et al.*, 1984; Dhokia *et al.*, 1986; Hayes *et al.*, 1985; Hilkens *et al.*, 1984, 1986; Kufe *et al.*, 1984). All of these monoclonal antibodies define antigens which have common biochemical characteristics: glycoproteins of high molecular weight ($M_r > 300,000$ by SDS-PAGE) with extensive O-linked carbohydrate side chains, i.e. mucin-like. In addition these monoclonal antibodies also react with human milk and human milk-fat globule membrane (HMFGM) and some antibodies have been shown to react with the PAS-O glycoprotein from human milk which is the one human tumour associated mucin glycoprotein bearing human tumour associated epitopes (Shimizu *et al.*, 1982; Ormerod *et al.*, 1985). The PAS-O component has also been referred to as EMA (epithelial membrane antigen) complex (Ormerod *et al.*, 1983, 1985) and recently popularised as human PEM (polymorphic epithelial mucin) (Taylor-Papadimitriou *et al.*, 1988) because of the genetic polymorphism exhibited.

The purpose of this study was to isolate 3E1.2 antigen (MSA) from the serum, ascitic fluid and breast cancer tissue of patients with breast cancer, define its biochemical structure and investigate its relationship to previously described breast cancer associated antigens. MSA was found to be a novel variant of the high molecular weight glycoproteins of human breast cancer, as monoclonal antibody 3E1.2 does not react with components of human milk.

### Materials and methods

### *Monoclonal antibodies*

Mouse monoclonal antibody 3E1.2 (anti-MSA) was used to immunopurify MSA in this study. The antibody is a pentameric IgM raised against human breast cancer cells derived from a primary carcinoma of the breast (Stacker *et al.*, 1985). Control antibodies used were 5C1 (anti-carcinoma of the colon), 3B11C8 (anti-carcinoma of the lung) and 676 (anti-human CD8) (produced in our laboratory). All of these antibodies were of the $\mu$ heavy chain isotype and were made in our laboratory. The following anti-human milk fat globule membrane antibodies were employed: HMFG-1 (IgG1), kindly supplied by Dr J. Taylor-Papadimitriou, ICRF, London (Taylor-Papadimitriou *et al.*, 1981), BC2 (IgG1), BC3 (IgM), 41.3 (IgM), 4B6.1 (IgM) (produced in our laboratory) and DF3 (IgG1) obtained from a CA 15-3 IRMA kit (Centocor, Malvern, PA, USA), supplied by the Australian Atomic Energy Commission (Kufe *et al.*, 1984). The IgM monoclonal antibodies were purified from ascitic fluid by lipid extraction using freon (1,1,2-trichloro-trifluorethane, Fluka AG, Buchs, Switzerland) and dialysis

Correspondence: I.F.C. McKenzie.

against 5 mM Tris-HCl pH 7.4 at 4°C. The precipitate formed was collected by centrifugation at 800*g* for 30 min and resuspended in phosphate buffered saline (PBS), pH 7.4. The IgG antibodies were purified on protein A-sepharose (Pharmacia Inc., Piscataway, NJ) as described (Ey *et al.*, 1978). Purified antibodies (3E1.2 and 4B6.1) were covalently bound to cyanogen bromide activated Sepharose 4B (Pharmacia Fine Chemicals, Uppsala, Sweden) at about 3 mg ml$^{-1}$ of swollen beads.

*Collection of serum samples, ascitic fluid and breast carcinoma tissue*

Serum was obtained from normal subjects and from patients with metastatic breast cancer, and stored at −70°C. Ascitic fluid and primary breast carcinoma tissue were also obtained from one patient who had high circulating MSA level (>10,000 IU). Ascitic fluid was centrifuged at 10,000*g* for 10 min to pellet cell debris and the supernatant stored at −70°C.

*Preparation and deglycosylation of human milk fat globule membrane (HMFGM) and purification of the milk mucin*

Crude HMFGM were prepared from fresh human milk (Jarasch *et al.*, 1977) after ultracentrifugation at 100,000*g* for 1.5 h at 4°C using Beckman L8-70 with an SW-28 rotor. The pellet was resuspended in 0.3 M sucrose, 70 mM $KCl_2$, 2 mM $MgCl_2$, 10 mM Tris-HCl buffer pH 7.4 and stored at −70°C. O-linked carbohydrate residues were removed from the HMFGM glycoproteins by treatment with trifluoromethanesulfonic acid (TFMSA) for 1 h at RT (room temperature) to produce deglycosylated HMFGM (Edge *et al.*, 1981). The milk mucin was purified from human skimmed milk by passage through a 4B6.1 affinity column followed by size exclusion chromatography (Burchell *et al.*, 1987); the high molecular weight fractions eluted were pooled and designated PAS-O (Shimizu *et al.*, 1982).

*Serological assays*

MSA activity in samples was determined by a competitive enzyme immunoassay (MSA inhibition assay) (Stacker *et al.*, 1987: Tjandra *et al.*, 1988) In brief, serum and affinity purified fractions of MSA were used to inhibit the binding of monoclonal antibody 3E1.2 to a membrane preparation of ZR75 breast cancer cells which contains MSA, and the results expressed as arbitrary inhibition units (IU) on a scale from 1 to 10,000 IU. Patients with advanced breast cancer have levels of 300–10,000 IU; 98% of normal individuals have low levels (<300 IU). In addition, the anti-HMFG activity of the monoclonal antibodies were evaluated in a HMFG binding assay wherein crude HMFGM were coated on to 96-well flexible PVC plates (Costar) at 25 $\mu$g ml$^{-1}$ in carbonate buffer pH 9.6 for 1 h at 37°C; non-specific binding sites were blocked with 1% BSA for 1 h at 37°C, and the plates washed with PBS pH 7.4/0.05% Tween 20. Purified antibodies (240 ng ml$^{-1}$ to 30 $\mu$g ml$^{-1}$) were then reacted with the solid phase antigens for 2 h at 37°C. After removal of excess antibody, sheep anti-mouse immunoglobulin conjugated to horseradish peroxidase (Amersham) was incubated for 3 h at 37°C. The plates were washed again, and developed using 0.03% ABTS (2.2-azino-di-[3-ethyl-benzthiazoline] sulphonate)/0.02% $H_2O_2$ in 0.1 M citrate buffer pH4 and absorbance read at 405 nm using an enzyme immunoassay plate reader (Titertek Multiscan MC). The reactivity of antibodies to deglycosylated HMFGM was similarly determined using wells coated with deglycosylated HMFGM and the bound antibody detected as for the HMFGM binding assay. In addition, a sandwich immunoassay was used to assess the epitope expression on immunopurified MSA: hybridoma ascitic fluid (1/500 in PBS) was adsorbed to the wells of flexible PVC plates (Costar) at 10 $\mu$l well$^{-1}$ and after incubation at 4°C for 18 h, the wells were washed with washing buffer (PBS pH 7.4/0.05% Tween 20); aliquots of MSA (10 $\mu$l) or washing buffer alone were added to the wells and after incubation for 1 h at room temperature, the wells were washed, $^{125}$I-3E1.2 antibody (10$^5$ c.p.m. 10 $\mu$l$^{-1}$ well$^{-1}$) added and incubated for 1 h at room temperature. Finally, the wells were washed and the radioactivity in each well determined.

*Purification of 3E1.2 defined antigen (MSA) from breast carcinoma tissue, serum and ascites*

Breast carcinoma tissue (15 g) was homogenised in PBS, pH 7.3 containing 5 mM $MgCl_2$ at 4 ml g tissue$^{-1}$ and an extranuclear membrane (ENM) preparation was isolated as the 105,000*g* pellets of 600*g* supernatants of the homogenate (Price *et al.*, 1985). The ENM extracts were obtained with 0.5% Nonidet P-40 (NP-40) in 0.1 M Tris-HCl, pH 7.0 for 30 min at 4°C and a soluble extract was obtained following centrifugation at 105,000*g* for 60 min. 3E1.2 defined antigen preparations were isolated from detergent (NP-40) solubilised subcellular membranes from breast carcinoma, serum containing high levels of MSA (>10,000 IU) and ascites by immunoaffinity chromatography using Sepharose-linked 3E1.2 antibodies. The samples were diluted 1:1 with PBS pH 7.6 containing 5 mM iodoacetamide, 2% aprotinin (Sigma Chemical Co., St Louis, MO, USA) and 0.02% $NaN_3$ and reacted with 3E1.2-Sepharose beads for 1 h at 4°C. After incubation the beads were packed in a 10 ml column and washed extensively with PBS pH 7.6 at 4°C. Bound MSA was eluted in 1 ml fractions with 1% diethylamine, pH 11.8 at room temperature (RT) and neutralised with 1 M Tris-HCl pH 7.0. Samples were then freeze dried and tested for activity using the inhibition assay. MSA was also isolated from serum on wheat germ agglutinin (WGA)-Sepharose 6MB, followed by elution with 0.7 mg ml$^{-1}$ *N*-acetyl glucosamine as described elsewhere (Gurd *et al.*, 1974).

*Sodium dodecyl sulphate-polyacrylamide gel electrophoresis (SDS-PAGE) and immunoblotting*

Samples were analysed by SDS-PAGE (Laemmli, 1970) and after electrophoresis gels were stained with either 0.2% Coomassie blue or 0.25% Croci en scarlet followed by extensive destaining (7% acetic acid for Coomassie blue, 7% acetic acid/12% ethanol for Croci en scarlet) or by Periodic acid-Schiff (PAS)-silver stain technique (Dubray *et al.*, 1982). Gels were then dried under vacuum, and autoradiographed at −70°C using XAR-5 film (Eastman Kodak, Rochester, NY, USA) and intensifying screens (DuPont, Wilmington, DE, USA). Molecular weight markers used were: 200,000 myosin, 116,000 $\beta$-galactosidase, 92,500 phosphorylase b, 66,000 bovine serum albumin, 43,000 ovalbumin (Biorad Laboratories). Monoclonal antibody immunoblotting analysis of samples (under reducing conditions) after fractionation by SDS-PAGE was performed as described elsewhere (Towbin *et al.*, 1979). Nitrocellulose membranes (Schleicher and Schuell, Dassel, FRG) were reacted with a 1/5 dilution of tissue culture supernatant containing the antibody and developed using sheep anti-mouse immunoglobulin conjugated to horseradish peroxidase (Amersham). Alternatively, blotted proteins were reacted with 200,000 c.p.m. of radiolabelled DF3, BC3 or control antibody and then autoradiographed.

*Radiolabelling of proteins and solid phase immunoprecipitation technique (SPIT)*

Iodination of purified MSA with Na$^{125}$I (Amersham International, Amersham, UK) was catalysed using Iodobeads (Pierce Chemical Co. Inc., Rockford, IL, USA) (Markwell, 1982). Crude HMFGM was radiolabelled, solubilised in 2% (w/w) sodium deoxylate, 8 M urea and 1% (v/v) 2-mercaptoethanol for 30 min at 37°C, then centrifuged at 10,000*g* for 20 min. Free $^{125}$I was removed from the supernatant by chromatography on Sephadex G-25 (PD-10,

Pharmacia) equilibrated in Tris-HCl buffer (pH 8.0) containing 100 mM NaCl and 10 mM sodium deoxycholate. In the SPIT assay, purified monoclonal antibodies were coated at $40\,\mu g\,ml^{-1}$, in a carbonate buffer pH 9.6, to 96-well flexible PVC plates (Costar, Cambridge, MA, USA) for 2 h at 37°C. Excess antibody was removed by washing with PBS/0.05% Tween 20 and non-specific binding sites blocked by incubation with 2% normal mouse serum (NMS)/2% bovine serum albumin (BSA)/PBS for 2 h at 37°C. After washing, iodinated affinity purified MSA in 2% NMS/2% BSA/PBS pH 7.6 was added to the wells (~750,000 c.p.m. per well) and incubated overnight at 4°C. Following this, the solid phase was washed extensively with PBS/0.05% Tween 20. Plates were dried, cut, and counted in a gamma-counter. In some cases, radiolabelled MSA was eluted from the solid phase with 8 M urea/0.0625 M Tris/2.3% SDS/50 mM DTT (Dithiothreitol) and analysed by SDS-PAGE as described.

*Periodate and neuraminidase (NE) treatment*

Periodate oxidation was performed by treating ZR75 membrane preparation ($MSA^+$), which has been coated on microtitre plates (Tjandra *et al.*, 1988). Sodium meta-periodate (Ajax Chemicals, Australia) was added at concentrations ranging from 10 to 100 mM, in a buffer of 50 mM sodium acetate, pH 4.5 for 2 h at 4°C. Wells were treated with 1% BSA to block non-specific binding sites, and then tested for antibody 3E1.2 binding by ELISA as described before. The immunoperoxidase assays on sections of formalin fixed breast cancer tissues were performed as previously described (Stacker *et al.*, 1985). The tissue sections which were subjected to neuraminidase treatment were incubated with 1 IU per slide neuraminidase (from *Vibrio cholerae*, Institut Behring) for 2 h at 37°C, after they had been treated with 0.5% $H_2O_2$ in PBS to remove endogenous peroxidase activity. After neuraminidase treatment, the slides were washed, exposed to the monoclonal antibody (1:10 dilution of tissue culture supernatant of 1:1,000 diluted mouse ascites fluid) and processed.

*Gel filtration chromatography*

MSA isolated by affinity chromatography with either antibody 3E1.2 or WGA lectin was applied to a 90 × 2.5 cm Sepharose-6B column (Pharmacia), equilibrated in PBS/pH 7.6 and prewashed with 3.0 ml of human stable plasma protein solution (Commonwealth Serum Laboratories, Melbourne, Australia) at 20°C. Fractions of 1.0 ml were collected and MSA activity determined by the inhibition assay. The column was calibrated with molecular weight markers: blue dextran $2\times10^6$, thyroglobulin 669,000, aldolase 158,000 and chymotrypsinogen A 25,000 (Pharmacia).

*Protease digestion*

Radiolabelled MSA and PAS-O were immunopurified by 3E1.2-Sepharose and 4B6.1-Sepharose beads respectively, separated by 5% SDS-PAGE, and detected by autoradiography. The bands containing the relevant material were excised using a scalpel blade and reswelled in digestion buffer (10% (v/v) glycerol, 0.1% (w/v) SDS, 0.125 M Tris-HCl, pH 6.8). The swelled gel strips were homogenised and digested for 4 h at 37°C with the following enzymes at concentrations of $5–250\,\mu g\,ml^{-1}$: thermolysin (Sigma Chemical Co.), trypsin (Miles Laboratories, Eckhart, IN, USA), proteinase K (Boehringer Mannheim, FRG), *Staphylococcus aureus* V8 protease (CalBiochem-Behring, La Jolla, CA, USA), Pronase (Boehringer Mannheim) and subtilisin (Boehringer Mannheim). Digestions were also performed with 10 mM DTT. Fragments of gels were pelleted by centrifugation and aliquots of the supernatant containing the digested protein loaded on to 15% SDS gels and the peptides detected by autoradiography. In all cases radiolabelled BSA or immunoglobulin served as a protease susceptible control substrate.

### Results

*Isolation of MSA and analysis by SDS-PAGE and immunoprecipitation*

MSA was isolated fron the serum of patients with advanced breast cancer where it is present in a soluble form and in relatively large amounts. Serum samples were subjected to immunoaffinity chromatography with immobilised monoclonal antibody 3E1.2, and eluted antigen was analysed by SDS-PAGE, Coomassie blue staining and PAS-silver staining (Figure 1), and its activity determined by the inhibition assay. Although MSA stained poorly with Coomassie blue (Figure 1a), it stained well with PAS-silver stain (Figure 1b). Virtually all of the initial MSA activity was recovered from the antibody column in a single broad peak; the absence of extraneous bands in SDS-PAGE

**Figure 1** Coomassie blue staining (**a**) and PAS-silver staining (**b**) of 5% SDS-polyacrylamide gels, of serum with MSA level >10,000 IU (track A), affinity purified MSA (track B) and HMFGM (track C). Molecular weight markers are as indicated. S indicates stacking gel. Differences in the intensity of the high molecular weight bands between serum and affinity purified MSA in the PAS-silver stain could be accounted for by the difference in the amount loaded on to the gel.

analysis (Figure 1) indicated that the preparation was pure. This does not eliminate, however, the possibility that there may be contaminants with the same electrophoretic mobility as MSA. The fractions containing purified material were pooled, radiolabelled and assayed by a solid phase immunoprecipitation technique (SPIT) for activity with 3E1.2 and other isotype matched control antibodies. The 3E1.2 monoclonal antibody immune precipitated more than 20 times the amount of radiolabelled antigen precipitated by the control antibodies. When these samples were eluted with 8 M urea/SDS sample buffer from the coated wells and analysed by SDS-PAGE, two discrete bands with $M_r$ values of 300,000–400,000 were observed (Figure 2a). Identical patterns were seen under reducing and non-reducing conditions, indicating that the two molecules are not covalently linked. The 3E1.2 antibody was also reactive with the immunoadsorbent purified MSA from serum on immunoblots and Figure 2b (tracks D and E) show the reaction of antibody 3E1.2 with the dominant $M_r$ 300,000–400,000 bands of the MSA.

**Figure 2** Analysis of MSA by SPIT assay, SDS-PAGE, and immunoblots. In (**a**) fractions eluted from the immunoaffinity column that contained MSA activity were pooled, radiolabelled and used for SPIT assay using various antibodies. The bound MSA was eluted from the solid phase analysed by 5% SDS-PAGE. Track A, 3E1.2 (lysate reduced); track B, 3E1.2 (lysate non-reduced); track C, 5C1; track D, 3B11C8; and track E, 676. In (**b**) immunoblotting of ascites (track A), NP-40 solubilised breast cancer tissue (track B), serum (track C) and 3E1.2 defined antigen (MSA) isolated from serum by immunoaffinity chromatography (tracks D and E) was performed; all samples from the same patient with advanced breast cancer. Samples were separated by SDS-PAGE, electrophoretically transferred on to nitrocellulose paper and 'stained' with the 3E1.2 antibody. Molecular weight marker proteins are as shown.

*Reactivity of 3E1.2 antibody with serum, ascites and extranuclear membrane preparations*

Analysis of serum samples from a number of patients with advanced breast cancer by immunoblotting with 3E1.2 showed a heterogeneous expression of MSA (Figure 3). Three basic variations were seen: (a) a predominant single band of approximate $M_r$ 380,000 (Figure 3, patient A); (b) both upper ($M_r$ 380,000) and lower ($M_r$ 330,000) bands (Figure 3, patients B and C); (c) a single lower band ($M_r$ 330,000) only (Figure 3, patient D). This immunoblotting experiment demonstrates that the epitope for 3E1.2 is expressed on both $M_r$ species (Figure 3, patients B and C). A smaller molecular weight species ($M_r$ 70,000–100,000) was also detected in some patients on immunoblot of SDS-PAGE-separated serum (Figures 2b and 3), indicating that the lower molecular weight material also expresses an epitope reactive with antibody 3E1.2 and may share a functional as well as immunological relationship with the high molecular weight material on which many cancer-related determinants are expressed later. However, the absence of the low molecular weight species ($M_r$ 70,000–100,000) in the immunopurified MSA on immunoblot (Figure 2b) suggests that they do not bind to 3E1.2 immunoaffinity column to any significant extent (Figure 3, tracks D and E); the reason of which is not entirely clear but it may indicate that the low molecular weight species are not exposed in the native state, in the absence of SDS but this is not proved.

The ascites and ENM preparations from breast carcinoma tissue of a patient with advanced breast cancer who had a high circulating MSA level (>10,000) was analysed by the inhibition assay. Both the ascites and ENM preparations and strong inhibitory activity (8,000 and 5,000 IU respectively). The nature of the 3E1.2 defined antigen (MSA) in serum, ascites and ENM preparations of the same patient was examined by Western blot analysis with 3E1.2 antibody probe. As shown in Figure 2b, the 3E1.2 defined antigen (MSA) was identified as a diffusely migrating band of similar high apparent molecular weight (>300,000), irrespective of the source of the antigen in the same patient. The pattern was reproducible on repeated testing with specimens from other patients and is similar to that obtained with immunoadsorbent purified MSA from serum when probed with 3E1.2 antibody (Figure 2b). A faint band of 3E1.2 reactive material of $M_r \sim$ 220,000 was also noted in the ENM preparation from breast carcinoma tissue.

Clearly, 3E1.2 defined antigen (MSA) present in detergent solubilised tumour membrane preparations were similar to 3E1.2 defined antigen present in serum and ascites of the same patient. Similar high molecular weight components reactive with 3E1.2 antibody in normal sera were not easily identified by immunoblotting (data not shown).

**Figure 3** Reactivity of monoclonal antibody 3E1.2 with serum samples from selected patients with metastatic breast cancer was studied. Sample E was the residual serum sample after mixing serum sample F with 3E1.2 sepharose beads. Serum (5 $\mu$l) was subjected to 5% SDS-PAGE analysis, transferred to nitrocellulose paper and probed with 3E1.2. Approximate molecular weight standards are indicated.

*Biochemical analysis of MSA*

To determine whether carbohydrate structures are required for binding of the antibodies, the effects of sodium periodate treatment on antibody binding were tested. Results shown in Figure 4 indicate that the epitope detected by 3E1.2 was sensitive to the periodate treatment, suggesting that carbohydrate plays a role in the binding site of 3E1.2. The effect of neuraminidase treatment on the expression of the antigen in breast cancer tissues was also studied. The binding of 3E1.2 antibody was markedly diminished by neuraminidase treatment in all five formalin fixed breast cancer tissues tested, as assessed by the percentage of carcinoma cells stained and the intensity of staining on immunoperoxidase assay (Figure 5). By contrast, the binding of BC3 antibody (anti-HMFG) was enhanced by neuraminidase treatment in all five breast carcinomas (Figure 5); the binding of BC2 antibody (anti-HMFG) was similarly enhanced by neuraminidase treatment (data not shown). These results suggest

**Figure 4** Effect of periodate on the 3E1.2 defined antigen (MSA). Membrane preparations of ZR75 breast cancer cells were treated with (A) PBS; (B) sodium acetate buffer pH 4.5; or periodate; (C) 10 mM, (D) 30 mM, (E) 50 mM. The reactivities of the treated preparations with antibody 3E1.2 were then tested in an ELISA binding assay. (A) and (B) serve as controls.

that sialic acid is required for binding of 3E1.2 antibody, and epitopes for BC2 and BC3 antibodies (anti-HMFG) were further exposed by removal of sialic acid.

*Analysis of MSA by gel filtration chromatography*

3E1.2 antigen (MSA) has been found to bind the lectin WGA (wheat germ agglutinin) and the MSA activity of fractions eluted with *N*-acetyl glucosamine determined in the MSA inhibition assay (data not shown). The MSA isolated from serum by 3E1.2 immunoaffinity chromatography and WGA lectin affinity chromatography was subjected to gel filtration chromatography on a Sepharose-6B column to determine its $M_r$ in the absence of SDS. Fractions eluted from the column were analysed for their reactivity with antibody 3E1.2 by the inhibition assay; using either antibody 3E1.2 or lectin affinity chromatography, the activity was confined to the material (MSA) eluted between the molecular weight markers blue dextran ($2 \times 10^6$) and thyroglobulin (669,000). This suggests that stable MSA aggregates exist under non-denaturing conditions and such aggregation may confer stability to the MSA molecule. In addition, the binding of MSA to lectin WGA suggests that MSA contains exposed *N*-acetylneuraminic acid and $\beta$(1-4)-*N*-acetyl glucosamine (Alles *et al.*, 1973; Nagata *et al.*, 1974)

*Binding of 3E1.2 to components of human milk*

Given that many other monoclonal antibodies to high molecular weight breast cancer associated antigens cross-react with components of human milk, it was important to determine if the epitope detected by 3E1.2 was also expressed on components of human milk. A binding experiment with HMFGM (Figure 6a) showed that antibody 3E1.2 and a control antibody (5C1) had no or minimal reactivity with HMFGM, but antibodies HMFG-1 and BC3, known to detect a high molecular weight glycoprotein if HMFGM (Burchell *et al.*, 1983; unpublished observations), were found to bind at a much lower concentration than 3E1.2. Since the cross-reactive molecule in HMFGM has also been reported in skimmed milk, samples of fresh whole milk were analysed by SDS-PAGE and tested by immuno-

**Figure 5** Sections of breast carcinoma stained by immunoperoxidase staining with antibodies 3E1.2 or BC3. Counterstained with haematoxylin (×100). (**a**) without neuraminidase (NE) treatment; (**b**) with NE treatment.

blotting (Figure 6b). Antibody 3E1.2 showed no detectable reactivity with any components of human milk (Figure 6b, track A), but antibody 41.3 (anti-HMFG) detecting the high molecular weight glycoprotein gave a clear reactivity with these components (Figure 6b, track B). To determine if the epitope detected by 3E1.2 was masked by carbohydrate on HMFGM, deglycosylated HMFGM glycoproteins were prepared and tested in the solid phase binding assay. 3E1.2 was non-reactive with both glycosylated HMFGM and deglycosylated HMFGM; in contrast, antibody BC3 (IgM), known to detect a high molecular weight glycoprotein of HMFGM, bound to both glycosylated and deglycosylated HMFGM (full data not shown). Similarly, the reactivity of antibody HMFG-1 with both glycosylated and deglycosylated HMFGM had been previously noted (Burchell *et al.*, 1987). These experiments clearly show the absence of the epitope detected by monoclonal antibody 3E1.2 on components of human milk. However, as antibody 3E1.2 bound poorly to milk-derived mucin (Figure 6), rigorous testing of whether antibody 3E1.2 binds core protein may require purification and deglycosylation of mucin from sources which react with the 3E1.2 antibody.

### *Digestion of MSA and PAS-O with proteases*

Limited proteolytic digestion of SDS-PAGE purified MSA with a panel of proteases showed that the molecules detected by antibody 3E1.2 were resistant to digestion with trypsin, *Staphylococcus aureus* V8 protease and thermolysin but sensitive to proteinase K (Figure 7a), pronase and subtilisin

**Figure 6** Reactivity of monoclonal antibody 3E1.2 with components of human milk. (**a**) Solid phase binding study showing binding of HMFG-1, BC3, 3E1.2 and control antibody (5C1) to preparations of human milk fat globule membrane; (**b**) whole human milk was fractionated on a 5% SDS polyacrylamide gel, transferred to nitrocellulose and probed with monoclonal antibodies (A) 3E1.2, (B) 41.3 (anti-HMFG), (C) anti-CD8 antibody (negative control). Approximate molecular weight markers are indicated.

**Figure 7** Analysis of $^{125}$I-labelled MSA and PAS-O after proteolytic digestion by SDS-PAGE and autoradiography. (**a**) Gel purified MSA was subjected to limited proteolytic digestion with 10 $\mu$g ml$^{-1}$ of the following proteases: (A) trypsin, (B) *Staphylococcus aureus* V8 protease, (C) thermolysin and (D) proteinase K for 4 h at 37°C; (**b**) Gel purified BSA, MSA and PAS-O were subjected to limited proteolytic digestion with 5 $\mu$g ml$^{-1}$ of subtilisin and pronase. Identical amounts (c.p.m.) of radiolabelled MSA and PAS-O were loaded in each track. Molecular weight markers, shown as daltons × 10$^3$, are as follows: phosphorylase b (97), bovine serum albumin (66), ovalbumin (43), carbonic anhydrase (31) and soybean trypsin inhibitor (22).

(Figure 7b). MSA was also subjected to digestion with a large excess of trypsin, *Staphylococcus aureus* V8 protease and thermolysin (250 $\mu g\,ml^{-1}$) for 16 h at 37°C with identical results (data not shown). To investigate the relationship between MSA and the high molecular weight glycoprotein of human milk (PAS-O), proteolytic digestion was performed using the enzymes pronase and subtilisin on gel purified MSA and PAS-O (Figure 7b). The subtilisin digest of MSA contained a cluster of glycopeptide bands of $M_r$ values 60,000–100,000. These bands were absent in the corresponding PAS-O channel. However, it cannot be confidently concluded that this disparity is due solely to differences in the polypeptide backbones of the two structures since differential glycosylation might also contribute to the different patterns of proteolytic digestion of MSA and PAS-O.

### *Multiple epitope expression on MSA*

MSA eluted from a 3E1.2 immunoaffinity column was tested for its reactivity with a number of monoclonal antibodies which detect epitopes known to be expressed on PAS-O of HMFGM and the high molecular weight glycoproteins seen in the serum of patients with breast cancer. Antibodies DF3 and BC3 (anti-HMFG) were tested for their reactivity on MSA by immunoblotting (Figure 8). Both antibodies reacted with MSA, indicating that their epitopes are present on some or all 3E1.2$^+$ molecules. Antibodies 41.3 and 4B6.1, directed to glycoprotein PAS-O of human milk also reacted with MSA (data not shown). These studies indicate that the MSA molecule contains a significant number of epitopes in common with PAS-O and the high molecular weight glycoprotein previously described in breast cancer.

As shown in Table I, using a sandwich immunoassay, when hybridoma ascitic fluids were adsorbed to the wells of PVC plates and then treated with MSA, purified from serum, there was subsequent significant binding of $^{125}$I-labelled 3E1.2 antibody only in wells coated with 3E1.2 and BC3 antibodies but not in wells coated with control antibody

**Figure 8** Immunoblotting of MSA obtained from the immunoaffinity column with antibodies DF3, BC3 and control antibody (5C1). Blots were autoradiographed as described. Molecular weight markers are indicated.

**Table I** Sandwich immunoassay for the analysis of epitopes on 3E1.2 defined antigen

| *Hybridoma ascitic fluid (1/500) adsorbed to wells* | *Binding of $^{125}$I-3E1.2 (mean c.p.m. ± s.d. × 10$^2$) bound to hybridoma ascitic fluid coated wells treated with* | |
|---|---|---|
| | *Buffer* | *MSA* |
| 3E1.2 | 1±1 | 52±4 |
| BC3 (anti-HMFG) | 2±0 | 28±3 |
| 5C1 (control antibody) | 2±0 | 3±1 |

(5C1). This indicates that the MSA expresses repeated 3E1.2 defined epitopes and the experiment confirms the co-expression of 3E1.2 and BC3 epitopes on at least a proportion of the MSA molecules.

### Discussion

Monoclonal antibody 3E1.2 was produced against a primary carcinoma of the breast, and detects a breast cancer associated antigen present in human serum called mammary serum antigen (MSA) (Stacker *et al.*, 1985; Tjandra *et al.*, 1988). Studies have shown that MSA is elevated in the sera of a high proportion of patients with breast cancer, compared to normal individuals, and that the levels are useful for monitoring patients with breast cancer (Stacker *et al.*, 1987; Tjandra *et al.*, 1988). Using the sera from patients with advanced breast cancer as a source of antigen, MSA was isolated using immunoaffinity chromatography and a solid phase immunoprecipitation technique. Subsequent SDS-PAGE and immunoblotting analysis showed the antigenic determinants for antibody 3E1.2 reside on heterogeneous molecules of $M_r$ 300,000–400,000. These molecules stained poorly with Coomassie blue but stained well in a PAS-silver stain and they bound the lectin WGA (data not shown), indicating the glycoprotein nature of MSA. The high molecular weight of MSA was confirmed by gel filtration chromatography of material eluted from a 3E1.2 affinity column and a WGA affinity column. There was a difference between the native ($M_r = 0.7$–$2.0 \times 10^6$) and apparent ($M_r \sim 300{,}000$) molecular weights. Whether the larger molecules arise by spontaneous association of monomers or through link proteins is not clear (Wesley *et al.*, 1985; Pearson *et al.*, 1981).

The results of the immunoblotting tests indicate that 3E1.2 defined antigen (MSA) from serum, ascites or breast carcinoma of the same patient displayed similar mobility in SDS-PAGE gels. In addition, the antigens purified from different sources had strong inhibitory activity when tested in the MSA inhibition assay. It appears that 3E1.2 defined antigen (MSA) can be released from breast carcinoma to the circulation and also locally into the ascites. The level of MSA in the circulation has been shown to depend on the tumour burden and tumour grade but is also dependent on some other factors as yet unidentified, as some cases of carcinoma *in situ* had elevated circulating MSA level (Tjandra *et al.*, 1988; Hare *et al.*, 1988).

Studies by other workers have also demonstrated the existence of high molecular weight glycoproteins on human breast cancer cells (Ashall *et al.*, 1982; Burchell *et al.*, 1984; Linsley *et al.*, 1986; Ormerod *et al.*, 1983; Papsidero *et al.*, 1984*a*; Price *et al.*, 1985; Sekine *et al.*, 1985) and in the serum of patients with breast cancer (Hayes *et al.*, 1985; Hilkens *et al.*, 1986; Burchell *et al.*, 1984; Linsley *et al.*, 1986; Papsidero *et al.*, 1984*b*). A number of these molecules are also related to the PAS-O glycoprotein found on the human milk fat globule and the epithelial membrane antigen (EMA) found in human milk (Shimizu *et al.*, 1982; Ormerod *et al.*, 1985), which is not surprising since many of the antibodies which define these breast cancer associated antigens were originally produced against components of human milk (Arklie *et al.*, 1981; Hilkens *et al.*, 1984). Apart from having a similar tissue distribution (restricted mainly to secretory epithelium), these high molecular weight breast cancer associated antigens share a number of common biochemical features. Firstly, these antigens are high molecular weight mucin-like glycoproteins, in which the majority of carbohydrate is joined to the peptide core by O-glycosidic linkages. The molecular weights were variously reported as >300,000, but in most cases in the region of 300,000–400,000 by SDS-PAGE analysis (Abe *et al.*, 1987). Secondly, the molecules have a high content of carbohydrate, indicated by poor staining with conventional protein stains, but strong staining with specific carbohydrate stains, and by the binding of specific lectins, usually WGA and peanut agglutinin (PNA) (Burchell *et al.*, 1983; Ormerod *et al.*, 1983; Sekine *et al.*, 1985). Thirdly, the molecules are resistant to digestion by many commonly used proteases, but digestion by pronase and subtilisin, proteases which catalyse the hydrolysis of a wide variety of peptide bonds, have been reported (Sekine *et al.*, 1985; Shimizu *et al.*, 1982). Finally, many of the monoclonal antibodies that define these molecules in patients with breast cancer react with the glycoproteins PAS-O or EMA (epithelial membrane antigen) in human milk which also possess these biochemical features. Thus, these breast cancer associated antigens and normal glycoproteins of human milk may constitute a family of related molecules.

Clearly, as part of this study it was important to determine the relationship of MSA to these high molecular weight glycoproteins. The biochemical characteristics of MSA are similar to those of the above mentioned molecules in a number of ways: similar high molecular weight glycoproteins ($M_r$ by SDS-PAGE>300,000), binding of WGA lectin and susceptibility to attack by subtilisin and pronase. It was also shown that monoclonal antibodies to PAS-O and other high molecular weight glycoproteins of breast cancer also react with MSA. This implies that a number of epitopes, presumably carbohydrate or carbohydrate–protein epitopes, are shared between PAS-O and MSA, and is evidence to suggest that PAS-O and MSA may possess similar or identical polypeptide backbones or distinct polypeptide chains with similar carbohydrate side chains. There are, however, several properties which suggest that monoclonal antibody 3E1.2 defines a unique epitope, in particular the lack of or limited binding of 3E1.2 to components of human milk, HMFGM and deglycosylated HMFGM. However, it has been shown that relative levels of binding of antibodies HMFG-1 and HMFG-2 varied between cell lines from normal and malignant breast epithelium, with the HMFG-2 epitope being expressed more strongly on tumour cell lines (Burchell *et al.*, 1983). Likewise, antibody W5 bound more strongly to W1 affinity-purified mucin from milk than to mucin purified from serum of breast cancer patients (Linsley *et al.*, 1986). These observations indicate that mucins from different sources may vary antigenically and could account for the lack of or limited binding of antibody 3E1.2 to components of human milk, HMFGM and deglycosylated HMFGM but strong binding to a tumour cell line (ZR75) derived mucin (data not shown). In contrast, antibody BC3 (anti-HMFG) bound more strongly to a purified milk-derived mucin and deglycosylated HMFGM than to the tumour cell line (ZR75) derived mucin (data not shown), suggesting that core protein epitopes may be masked, modified or otherwise different in mucin glycoproteins from different sources (Burchell *et al.*, 1987). In addition, proteolytic digestion studies of MSA and PAS-O further suggest the molecular non-identity of these molecules. However, the effects of different degrees of glycosylation and the size or charge of peptide fragments and the accessibility of proteases preclude the firm interpretation that the differences between the molecules occur at the level of their respective polypeptide backbones. Therefore, at the very least, antibody 3E1.2 defines a unique epitope on a high molecular weight glycoprotein present in serum, ascites and breast carcinoma tissue but absent or present in very low amounts in milk. In addition, the reduced binding of antibody 3E1.2 upon periodate oxidation suggests that the epitope defined by antibody 3E1.2 involves carbohydrate. Since periodate oxidation of the antigen did not completely abolish the binding of antibody 3E1.2, a contribution by the protein backbone to the epitope cannot be excluded. Because of the high degree of glycosylation of mucin glycoproteins, it seemed likely that epitopes for many mucin directed antibodies would contain oligosaccharide structures. In addition, binding of 3E1.2 antibody was neuraminidase sensitive, suggesting that sialic acid is required for binding of this antibody.

The heterogeneity seen with these high molecular weight glycoproteins indicates that they constitute a family of related molecules. Recent studies by other workers (Griffiths *et al.*, 1987; Ormerod *et al.*, 1985; Price *et al.*, 1985, 1986) have shown that epitopes for a number of monoclonal antibodies reactive with high molecular weight glycoproteins of breast cancer are present on antigen preparations purified by monoclonal antibodies NCRC-11 and HMFG-2 respectively. In addition, antigen purified from human breast tumour cells and human milk by antibody DF3 has been shown to bind other monoclonal antibodies F36/22 and Ca1 (Abe *et al.*, 1987); further evidence for a family of related high molecular weight tumour associated glycoproteins. In this study, antibodies DF3 and BC3 were shown to bind purified MSA, thereby confirming a relationship between MSA and the other high molecular weight glycoproteins of human milk and breast cancer. Antibody DF3 is now known to react with a core protein epitope (Siddiqui *et al.*, 1988) and has been used to clone the same cDNA as was cloned by Gendler *et al.* (1988) using other antibodies (HMFG-1, HMFG-2 and SM-3). These data indicate that there is clearly a family of related molecules, each of which probably has the same core protein. Thus, it is possible that the 3E1.2 defined antigen (MSA) shares the same or similar core protein as DF3 defined antigen. It has been shown indirectly for the polymorphic epithelial mucin or PEM (Burchell *et al.*, 1983) and directly for the DF3 purified antigen (Hull *et al.*, 1988) that the glycosylation is different in normal (the carbohydrate component was analysed by Shimizu & Yamauchi (1982)) and cancer associated mucin. Thus both core protein and carbohydrate epitopes can be identified on the cancer associated PEM mucin which are not found, or to a much lesser degree on the normally processed mucin as in HMFGM. It appears that oligosaccharide structures are important components of the 3E1.2 defined epitope which is closely tumour related. Determining the exact relationship of these molecules will involve further studies examining the primary polypeptide structure and oligosaccharide moieties of this group of molecules.

Polymorphism was also seen in the MSA molecules obtained from different patients (Figure 3), as shown by the difference in mobilities of 3E1.2 reactive high molecular weight components in polyacrylamide gels. These differences could be due to either genetically determined allelic differences in the protein or carbohydrate portions of the molecules, or by the modification of sugar residues after cellular expression or secretion. In this context we note recent studies describing the genetic polymorphism of a urinary mucin-type glycoprotein related to the mucin glycoprotein of mammary tumours and human milk which defines a gene locus (PUM), with at least ten alleles which determine the expression of ten defined specificities (Gendler *et al.*, 1987; Swallow *et al.*, 1987). In the light of the relationship between MSA and the high molecular weight glycoproteins of human milk, a similar system could control the expression of MSA.

In summary, this study has shown that monoclonal antibody 3E1.2 defines a high molecular weight glycoprotein (MSA) in the serum, ascites and breast cancer tissue of patients with breast cancer. The 3E1.2 defined antigen

(MSA) appears to be related to a discrete family of high molecular weight glycoproteins defined in breast cancer patients sharing a number of common biochemical features and antigenic epitopes; however, monoclonal antibody 3E1.2 detects an epitope not expressed by PAS-O of human milk, making it a unique variant.

The authors would like to thank Ruth Godding, Mimi Morgan, Toula Athanasiadis and Jez McLaughlin for their assistance in preparation of this manuscript. We also thank Geoff Chambers and Melinda Lowe for their technical assistance, and Damian Purcell for the stimulating discussion.

### References

ABE, M. & KUFE, D. (1987). Identification of a family of high molecular weight tumor-associated glycoproteins. *J. Immunol.*, **139**, 257.

ALLEN, A.K., NUEBERGER, A. & SHARON, N. (1973). The purification, composition and specificity of wheat-germ agglutinin. *Biochem. J.*, **131**, 155.

ARKLIE, J., TAYLOR-PAPADIMITRIOU, J., BODMER, W.F., EGAN, M. & MILLIS, R. (1981). Differentiation antigens expressed by epithelial cells in the lactating beast are also detectable in breast cancers. *Int. J. Cancer*, **28**, 22.

ASHALL, F., BRAMWELL, M.E. & HARRIS, H. (1982). A new marker for human cancer cells. I. The Ca antigen and the Ca1 antibody. *Lancet* **ii**, 1.

BURCHELL, J., DURBIN, H. & TAYLOR-PAPADIMITRIOU, J. (1983). Complexity of expression of antigenic determinants recognized by monoclonal antibodies HMFG-1 and HMFG-2, in normal and malignant human mammary epithelial cells. *J. Immunol.*, **131**, 508.

BURCHELL, J., GENDLER, S., TAYLOR-PAPADIMITRIOU, J. & 4 others (1987). Development and characterization of breast cancer reactive monoclonal antibodies directed to the core protein of the human milk mucin. *Cancer Res.*, **47**, 5476.

BURCHELL, J., WANG, D. & TAYLOR-PAPADIMITRIOU, J. (1984). Detection of the tumour associated antigens recognized by the monoclonal antibodies HMFG 1 and 2 in serum from patients with breast cancer. *Int. J. Cancer*, **34**, 763.

DHOKIA, B., PECTASIDES, D., SELF, C. & 5 others (1986). A low pH enzyme linked immunoassay using two monoclonal antibodies for the serological detection and monitoring of breast cancer. *Br. J. Cancer*, **54**, 885.

DUBRAY, G. & BEZARD, G. (1982). A highly sensitive periodic acid-silver stain for 1,2-diol groups of glycoproteins and polysaccharides in polyacrylamide gels. *Anal. Biochem.*, **119**, 325.

EDGE, A.S.B., FALTYNEK, C.R., HOF, L., REICHET, L.E. & WEBER, P. (1981). Deglycosylation of glycoproteins by trifluoromethanesulfonic acid. *Anal. Biochem.*, **118**, 131.

ELLIS, I.O., ROBINS, R.A., ELSTON, C.W., BLAMEY, R.W., FERRY, B. & BALDWIN, R.W. (1984). A monoclonal antibody, NCRC-11, raised to human breast carcinoma. 1. Production and immunohistological characterization. *Histopathology*, **8**, 501.

EY, P.L., PROWSE, S.J. & JENKIN, C.R. (1978). Isolation of pure $IgG_1$, $IgG_{2a}$ and $IgG_{2b}$ immunoglobulins from mouse serum using protein A-Sepharose. *Immunochemistry*, **15**, 429.

GENDLER, S.J., BURCHELL, J.M., DUHIG, R. & 4 others (1987). Cloning a cDNA coding for differentiation and tumor-associated mucin glycoprotein expressed by human mammary epithelium. *Proc. Natl Acad. Sci. USA*, **84**, 6060.

GENDLER, S., TAYLOR-PAPADIMITRIOU, J., DUHIG, T., ROTHBARD, J. & BURCHELL, J. (1988). A highly immunogenic region of a human polymorphic epithelial mucin expressed by carcinomas is made up of tandem repeats. *J. Biol. Chem.* (in the press).

GRIFFITHS, A.B., BURCHELL, J., GENDLER, S. & 4 others (1987). Immunological analysis of mucin molecules expressed by normal and malignant mammary epithelial cells. *Int. J. Cancer*, **40**, 319.

GURD, J.W. & MAHLER, H.R. (1974). Fractionation of synaptic plasma membrane glycoproteins by lectin affinity chromatography. *Biochemistry*, **13**, 5193.

HARE, W.S.C., TJANDRA, J.J., RUSSEL, I.S., COLLINS, J.P. & McKENZIE, I.F.C. (1988). Comparison of mammary serum antigen (MSA) assay with mammography in patients with breast cancer. *Med. J. Aust.*, **149**, 402.

HAYES, D.F., SEKINE, H., OHNO, T., ABE, M., KEEFE, K. & KUFE, D.W. (1985). Use of a murine monoclonal antibody for detection of circulating plasma DF3 antigen levels in breast cancer patients. *J. Clin. Invest.*, **75**, 1671.

HILKENS, J., BUIJS, F., HILGERS, J. & 4 others (1984). Monoclonal antibodies against human milk fat globule membranes detecting differentiation antigens of the human mammary gland and its tumours. *Int. J. Cancer*, **34**, 197.

HILKENS, J., KROEZEN, V., BONFRER, J.M.G., DE JONG-BAKKER, M. & BRUNING, P.F. (1986). MAM-6, a new serum marker for breast cancer monitoring. *Cancer Res.*, **46**, 2582.

HULL, S.R., BRIGHT, A., CARRAWAY, K.L., ABE, M. & KUFE, D. (1988). Oligosaccharides of the DF3 antigen of the BT-20 human breast carcinoma cell line. *J. Cell Biochem.*, Suppl., 12E, T110.

JARASCH, E., BRUDER, G., KEENAN, T.W. & FRANKE, W.W. (1977). Redox constituents in milk fat globule membranes and rough endoplasmic reticulum from lactating mammary gland. *J. Cell Biol.*, **73**, 223.

KUFE, D., INGHIRAMI, G., ABE, M., HAYES, D., JUSTI-WHEELER, H. & SCHLOM, J. (1984). Differential reactivity of a novel monoclonal antibody (DF3) with human malignant versus benign breast tumours. *Hybridoma*, **3**, 223.

LAEMMLI, U.K. (1970). Cleavage of structural proteins during the assembly of the head of bacteriophage T4. *Nature*, **227**, 680.

LINSLEY, P.S., OCHS, V., LASKA, S. & 4 others (1984). Elevated levels of a high molecular weight antigen detected by antibody W1 in sera from breast cancer patients. *Cancer Res.*, **46**, 5444.

MARKWELL, M.A.K. (1982). A new solid-state reagent to iodinate proteins. 1. Conditions for the efficient labeling of antiserum. *Anal. Biochem.*, **125**, 427.

NAGATA, Y. & BURGER, M.M. (1974). Wheat germ agglutinin. Molecular characteristics and specificity for sugar binding. *J. Clin. Invest.*, **53**, 3116.

ORMEROD, M.G., McILHINNEY, J., STEELE, K. & SHIMIZU, M. (1985). Glycoprotein PAS-O from the milk fat globule membrane carries antigenic determinants for epithelial membrane antigen. *Mol. Immunol.*, **22**, 265.

ORMEROD, M.G., STEELE, K., WESTWOOD, J.H. & MAZZINI, N.M. (1983). Epithelial membrane antigen: partial purification, assay and properties. *Br. J. Cancer*, **48**, 533.

PAPSIDERO, L.D., CROGHAN, G.A., O'CONNELL, M.J., VALENZUELA, L.A., NEMOTO, T. & CHU, T.M. (1983). Monoclonal antibodies (F36/22 and M7/105) to human breast carcinoma. *Cancer Res.*, **43**, 1741.

PAPSIDERO, L.D., CROGHAN, G.A., JOHNSON, E.A. & CHU, T.M. (1984*a*). Immunoaffinity isolation of ductal carcinoma antigen using monoclonal antibody F36/22. *Mol. Immunol.*, **21**, 955.

PAPSIDERO, L.D., NEMOTO, T., CROGHAN, G.A. & CHU, T.M. (1984*b*). Expression of ductal carcinoma antigen in breast cancer sera as defined using monoclonal antibody F36/22. *Cancer Res.*, **44**, 4653

PEARSON, J.P., ALLEN, A. & PARRY, S. (1981). A 70,000 molecular weight protein isolated from purified pig gastric mucus glycoprotein by reduction of disulphide bridges and its implication in the polymeric structure. *Biochem. J.*, **197**, 155.

PRICE, M.R., EDWARDS, S., OWAINATI, A. & 4 others (1985). Multiple epitopes on a human breast carcinoma associated antigen. *Int. J. Cancer*, **36**, 567.

PRICE, M.R., EDWARDS, S., POWELL, M. & BALDWIN, R.W. (1986). Epitope analysis of monoclonal antibody NCRC-11 defined antigen isolated from human ovarian and breast cancer carcinomas. *Br. J. Cancer*, **54**, 393.

SEKINE, H., OHNO, T. & KUFE, D.W. (1985). Purification and characterization of a high molecular weight glycoprotein detectable in human milk and breast carcinomas. *J. Immunol.*, **135**, 3610.

SHIMIZU, M. & YAMAUCHI, K. (1982). Isolation and characterization of mucin-like glycoprotein in human milk fat globule membrane. *J. Biochem.*, **91**, 515.

SIDDIQUI, J., ABE, M., HAYES, D., SHANI, E., YUNIS, E. & KUFE, D. (1988). Isolation and sequencing of a cDNA coding for the human DF3 breast carcinoma-associated antigen. *Proc. Natl Acad. Sci. USA*, **85**, 2320.

STACKER, S.A., THOMPSON, C.H., RIGLAR, C. & McKENZIE, I.F.C. (1985). A new breast carcinoma antigen defined by a monoclonal antibody. *J. Natl Cancer Inst.*, **75**, 801.

STACKER, S.A., SACKS, N.P.M., THOMPSON, C.H. & 6 others (1987). A serum test for the diagnosis and monitoring of the progress of breast cancer. In *Immunological Approaches to the Diagnosis and Therapy of Breast Cancer,* Ceriani. R.L. (ed) p. 217. Plenum Press: New York.

SWALLOW, D.M., GENDLER, S., GRIFFITHS, B., CORNEY, G., TAYLOR-PAPADIMITRIOU, J. & BRAMWELL, M.E. (1987). The human tumour-associated epithelial mucins are coded by an expressed hypervariable gene locus *PUM. Nature,* **328,** 82.

TAYLOR-PAPADIMITRIOU, J.. PETERSON, J., ARKLIE, J., BURCHELL, J., CERIANI, R.L. & BODMER, W. (1981). Monoclonal antibodies to epithelium-specific components of the human milk fat globule membrane: production and reaction with cells in culture. *Int. J. Cancer,* **28,** 17.

TAYLOR-PAPADIMITRIOU, J., & GENDLER, S.J. (1988). Molecular aspects of mucins. *Cancer Rev.* (in the press).

THOMPSON, C.H., LICHTENSTEIN, M., STACKER, S.A. & 4 others (1984). Immunoscintigraphy for detection of lymph node metastases from breast cancer. *Lancet* **i,** 245.

TJANDRA, J.J., RUSSELL, I.S., COLLINS, J.P., STACKER, S.A. & McKENZIE, I.F.C. (1988). The application of mammary serum antigen assay in the management of breast cancer – a preliminary report. *Br. J. Surg.,* **75,** 811.

TOWBIN, H., STAEHELIN, T. & GORDON, J. (1979). Electrophoretic transfer of proteins from polyacrylamide gels to nitrocellulose sheets. *Proc. Natl Acad. Sci. USA,* **76,** 4350.

WESLEY, A., MANTLE, M., MAN, D., QURESHI, R., FORSTNER, G. & FORSTNER, J. (1985). Neutral and acidic species of human intestinal mucin. Evidence for different core peptides. *J. Biol. Chem.,* **260,** 7955.